# Diagnostic accuracy of a large language model in rheumatology: comparison of physician and ChatGPT-4

**DOI:** 10.1007/s00296-023-05464-6

**Published:** 2023-09-24

**Authors:** Martin Krusche, Johnna Callhoff, Johannes Knitza, Nikolas Ruffer

**Affiliations:** 1 Division of Rheumatology and Systemic Inflammatory Diseases, University Hospital Hamburg-Eppendorf (UKE), Hamburg, Germany; 2 Epidemiology Unit, German Rheumatism Research Centre, Berlin, Germany; 3 Institute for Social Medicine, Epidemiology and Health Economics, Charité Universitätsmedizin, Berlin, Germany; 4 Institute of Digital Medicine, University Hospital of Giessen and Marburg, Philipps University Marburg, Marburg, Germany; 5 Université Grenoble Alpes, AGEIS, Grenoble, France

**Keywords:** Large language models, ChatGPT, Rheumatology, Triage, Diagnostic process, Artificial intelligence

## Abstract

Pre-clinical studies suggest that large language models (i.e., ChatGPT) could be used in the diagnostic process to distinguish inflammatory rheumatic (IRD) from other diseases. We therefore aimed to assess the diagnostic accuracy of ChatGPT-4 in comparison to rheumatologists. For the analysis, the data set of Gräf et al. (2022) was used. Previous patient assessments were analyzed using ChatGPT-4 and compared to rheumatologists’ assessments. ChatGPT-4 listed the correct diagnosis comparable often to rheumatologists as the top diagnosis 35% vs 39% (*p* = 0.30); as well as among the top 3 diagnoses, 60% vs 55%, (*p* = 0.38). In IRD-positive cases, ChatGPT-4 provided the top diagnosis in 71% vs 62% in the rheumatologists’ analysis. Correct diagnosis was among the top 3 in 86% (ChatGPT-4) vs 74% (rheumatologists). In non-IRD cases, ChatGPT-4 provided the correct top diagnosis in 15% vs 27% in the rheumatologists’ analysis. Correct diagnosis was among the top 3 in non-IRD cases in 46% of the ChatGPT-4 group vs 45% in the rheumatologists group. If only the first suggestion for diagnosis was considered, ChatGPT-4 correctly classified 58% of cases as IRD compared to 56% of the rheumatologists (*p* = 0.52). ChatGPT-4 showed a slightly higher accuracy for the top 3 overall diagnoses compared to rheumatologist’s assessment. ChatGPT-4 was able to provide the correct differential diagnosis in a relevant number of cases and achieved better sensitivity to detect IRDs than rheumatologist, at the cost of lower specificity. The pilot results highlight the potential of this new technology as a triage tool for the diagnosis of IRD.

**Supplementary Information:**

The online version contains supplementary material available at 10.1007/s00296-023-05464-6.

## Introduction

Recent diagnostic and therapeutic advances in rheumatology are still counterbalanced by a shortage of specialists [1] resulting in a significant diagnostic delay [2]. Early and correct diagnosis is, however, essential to prevent persistent joint damage.

In this context, artificial intelligence applications including patient-facing symptom checkers represent a field of interest and could facilitate patient triage and accelerate diagnosis [3, 4]. In 2022, we were able to show that the symptom-checker Ada had a significantly higher diagnostic accuracy than physicians in the evaluation of rheumatological case vignettes [5].

Currently, the introduction of large language models (LLM) such as ChatGPT has raised expectations for their use in medicine [6]. The impact of ChatGPT's arises from its ability to engage in conversations and its performance that is either close to or on par with human capabilities in various cognitive tasks [7]. For instance, Chat-GPT has achieved satisfactory scores on the United States Medical Licensing Examinations [8] and some authors suggest that LLM applications might be suitable for clinical, educational, or research environments [9, 10].

Interestingly, pre-clinical studies suggest that this technology could also be used in the diagnostic process [11, 12] to distinguish inflammatory rheumatic from other diseases.

We therefore aimed to assess the diagnostic accuracy of ChatGPT-4 in comparison to a previous analysis including physicians and symptom checkers regarding rheumatic and musculoskeletal diseases (RMDs).

## Methods

For the analysis, the data set of Gräf et al. [5] was used with minor updates to disease classification regarding the grouping of diagnoses. The assessments of the symptom-checker app were analyzed using ChatGPT-4 and compared to the previous assessment results of Ada and the diagnostic ranking of the blinded rheumatologists. ChatGPT-4 was instructed to name the top five differential diagnoses based on the available information of the Ada assessment (see Supplement 1).

All diagnostic suggestions were manually reviewed. If an Inflammatory rheumatic disease (IRD) was among the top three (D3) or top five suggestions (ChatGPT-4 D5), respectively, D3 and D5 were summarized as IRD-positive (even if non-IRD diagnoses were also among the suggestions). Proportions of correctly classified patients were compared between the different groups using McNemar’s test. Classification of inflammatory rheumatic disease (IRD) status was additionally assessed.

## Results

**Fig. 1:**
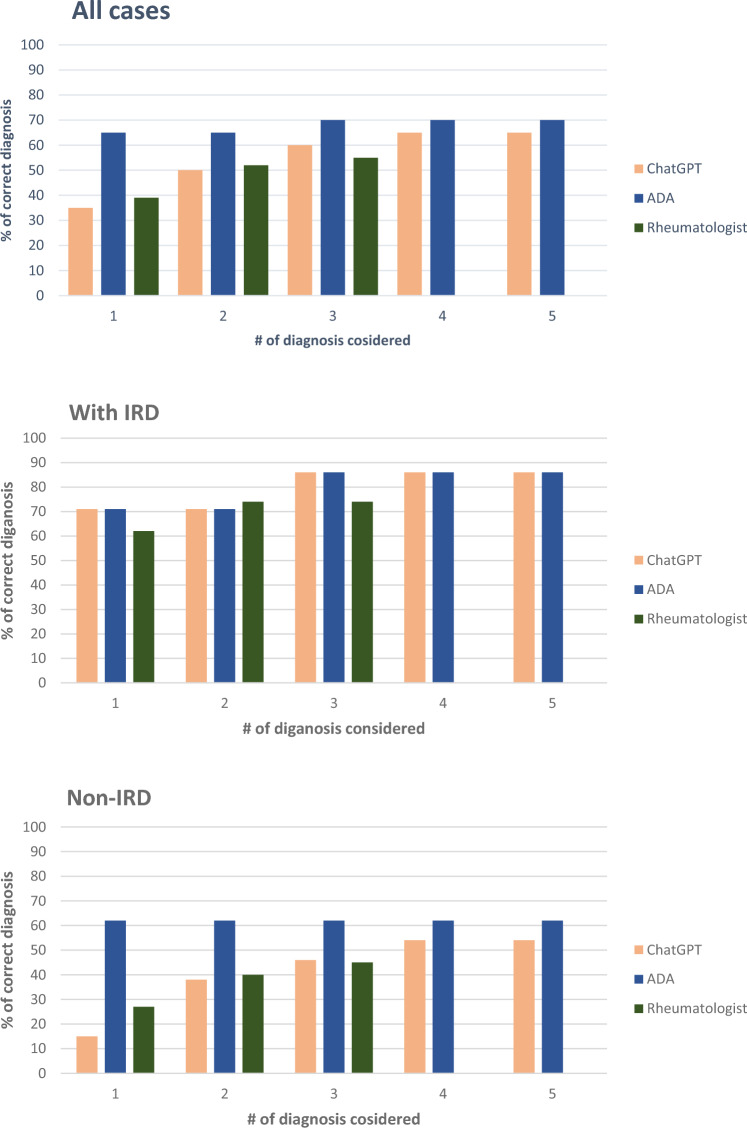
Percentage correctly classified diagnosis rank

ChatGPT-4 listed the correct diagnosis comparable often to physicians as the top diagnosis 35% vs 39% (*p* = 0.30); as well as among the top 3 diagnoses, 60% vs 55%, (*p* = *0.38)*. In IRD-positive cases, ChatGPT-4 provided the top diagnosis in 71% vs 62% in the physician analysis. The correct diagnosis was among the top 3 in 86% (ChatGPT-4) vs 74% (physicians). In non-IRD cases, ChatGPT-4 provided the correct top diagnosis in 15% vs 27% in the physician analysis. The correct diagnosis was among the top 3 in non-IRD cases in 46% of the ChatGPT-4 group vs 45% in the physician group (Fig. 1).

If only the first suggestion for diagnosis was considered, ChatGPT-4 correctly classified 58% of cases as IRD compared to 56% of the rheumatologists (*p* = 0.52). If the top 3 diagnoses were considered, ChatGPT-4 classified 36% of the cases correctly as IRD vs 52% of the rheumatologists (*p* = 0.01) (see Fig. 1). ChatGPT-4 had at least one suggestion of an inflammatory diagnosis for all non-IRD cases.

## Discussion

ChatGPT-4 showed a slightly higher accuracy (60% vs. 55%) for the top 3 overall diagnoses compared to the rheumatologist’s assessment. It had a higher sensitivity to determine the correct IRD status than rheumatologists, but considerably worse specificity, suggesting that ChatGPT-4 may be particularly useful for detecting IRD patients, where timely diagnosis and treatment initiation are critical. It could therefore potentially be used as a triage tool for digital pre-screening and facilitate quicker referrals of patients with suspected IRDs.

Our results are in line with those of Kanjee et al. [12] who demonstrated an accuracy of 64% for ChatGPT-4 evaluating the top 5 differential diagnoses of the *New England Journal of Medicine* clinicopathological conferences.

Interestingly, in the cross-sectional study of Ayers et al. [13], the authors found that chatbot responses to publicly asked medical questions on a public social media forum were preferred over physician responses and rated significantly higher for both quality and empathy, highlighting the potential of this technology as a first point of contact and source of information for patients. In summary, ChatGPT-4 was able to provide the correct differential diagnosis in a relevant number of cases and achieved better sensitivity to detect IRDs than a rheumatologist, at the cost of lower specificity.

Although this analysis has some shortcomings, i.e., the small sample size and the limited information (only access to the Ada assessments without further clinical data), it highlights the potential of this new technology as a triage tool that could support or even speed up the diagnosis of RMDs.

As digital self-assessment and remote care options are difficult for some patients due to limited digital health competencies [14], up-to-date studies should be conducted on how accurately patients can express their symptoms and complaints using AI and symptom-checker applications, so that we can benefit from these technologies more effectively.

Until satisfactory results are obtained, the use of artificial intelligence by GPs for effective referral instead of diagnostic use can be expanded and larger prospective studies are recommended to further evaluate the technology. Furthermore, issues, such as ethics, patient consent, and data privacy in the context of the use of artificial intelligence in medical-decision making, are crucial critical guidelines for the application of LLM technologies such as ChatGPT are needed [15].

### Supplementary Information

Below is the link to the electronic supplementary material.

Supplementary file1 (DOCX 32 KB)

## Data Availability

The datasets used and/or analysed during the current study are available from the corresponding author on reasonable request.
