# Supplementary material for: Diagnostic accuracy of a large language model in rheumatology: comparison of physician and ChatGPT-4
Source: Rheumatol Int. 2023 Sep 24;44(2):303–6. doi: 10.1007/s00296-023-05464-6 (PMC10796566; doi:10.1007/s00296-023-05464-6)
Supplement: Supplementary file 1 — Supplementary file1 (DOCX 32 KB) [file 296_2023_5464_MOESM1_ESM.docx]

**Supplementary Material 1**

ChatGPT-4 assessement

<https://chat.openai.com/share/7d1ea5ff-2a17-4aad-bf62-75d610173efd>

(assessed at the 10/07/2023)

German Assessment translated with www.DeepL.com/Translator (free version)

(assessed at the 20/08/2023):

*Which clinical diagnosis matches the following: Please provide the top 5 diagnoses*

*this is a female patient born in 1971*

*Present symptoms*

*pain in the metatarsal joints*

*has been present for: one month to one year*

*Movement: worsening*

*Laterality: bilateral*

*Intensity: severe*

*Reddened facial skin*

*persists since: more than one year*

*Pain: present*

*Pruritus: present*

*Dry skin of the eyelid*

*persists since: more than one year*

*Headache*

*Laterality: bilateral*

*throbbing: present*

*Preventing: aggravating*

*persists since: more than one year*

*Intensity: severe*

*Joint pain in fingers*

*existing since: more than one year*

*Movement: mitigating*

*Laterality: bilateral*

*Intensity: severe*

*Decreased mobility of the*

*metatarsophalangeal joint*

*Pressure painful*

*Metatarsophalangeal joint of big toe*

*Laterality: bilateral*

*Foot pain*

*during movement: without effect*

*Laterality: bilateral*

*Intensity: severe*

*Pain in the big toe*

*Morning stiffness*

*Pain in more than one*

*joint*

*Pain on exertion: alleviating*

*Fatigue*

*General muscle pain*

*Pressure pain of the*

*back muscles*

*Difficulty concentrating*

*Tingling sensation in the leg*

*Position: bilateral, asymmetrical*

*Swollen finger joint*

*Pressure painful finger joints*

*Laterality: bilateral*

*Symptoms not present are*

*Morning stiffness*

*Prolonged night sleep*

*Pressure painfulness of the*

*leg muscles*

*Diabetes*

*High blood pressure*

*Symptoms not present:*

*Lump under the skin at a*

*joint*

*Node under the skin on the*

*outside of the arm*

*Abdominal pain*

*Prolonged night sleep*

*Pressure pain in the lower*

*back muscles*

*Pregnant*

*Smoker*

*Diabetes*

*High blood pressure*

*Uncertain symptoms:*

*Swollen*

*Metatarsophalangeal joint of big toe*

*Pressure pain of the*

*Leg muscles*

*ChatGPT*

*Please note that an online AI cannot provide a medical diagnosis. The information provided here is for informational purposes only and is not a substitute for professional medical advice, diagnosis or treatment. It is always advisable to consult a physician or other health care provider if you have any health concerns.*

*Considering the symptoms described, the following conditions might be considered:*

*Rheumatoid arthritis: symptoms include joint pain and swelling, fatigue and morning stiffness, but described here as not present. However, it could be seronegative rheumatoid arthritis.*

*Psoriatic arthritis: pain and swelling in the joints, fatigue, and skin changes are common symptoms of this disease. The patient reports red skin and dry eyelids, which could be indicative of psoriasis.*

*Fibromyalgia: This could explain the generalized muscle pain, difficulty concentrating, and fatigue. However, joint swelling is not typical of fibromyalgia.*

*Gout: Pain and swelling, especially in the metatarsophalangeal joint, could indicate an attack of gout.*

*Systemic lupus erythematosus (SLE): this is an autoimmune disease that can cause joint pain, skin redness (especially on the face), and fatigue.*

*It is important that the patient receive a thorough evaluation by a medical professional to determine the exact cause of her symptoms. Further testing may also be needed to make an accurate diagnosis.*

*User*

*Which clinical diagnosis matches the following: Please provide the top 5 diagnoses*

*this is a female patient born in 1969*

*Present symptoms*

*Pale fingers*

*persists since: more than one year*

*Tingling or numbness in the hand*

*hand*

*Location: bilateral, symmetrical*

*Finger pain*

*during movement: without effect*

*Laterality: bilateral*

*Intensity: mild*

*Tingling or numbness in*

*ring and little finger*

*Pain in more than one*

*joint*

*Pain on exertion: ineffective*

*Extremely cold temperatures*

*exposed*

*Foot pain*

*during movement: without effect*

*Laterality: bilateral*

*Intensity: mild*

*Symptoms not present:*

*Cold finger*

*Blue finger*

*Swollen hand*

*Redness of the cheek skin*

*Blue toe*

*Difficulty swallowing*

*Muscle weakness in the leg*

*Rash on the face*

*Tingling or numbness in the*

*Foot*

*Reddened skin on the foot*

*Pain in the face*

*Swelling of the face*

*Blister on the face*

*Pain in toes*

*Reddened skin on hand*

*Pregnant*

*Smoker*

*Diabetes*

*High blood pressure*

*ChatGPT*

*The following conditions might be considered in light of the symptoms listed. Please note that this list is not exhaustive and requires professional medical evaluation and diagnosis.*

*Raynaud's Syndrome: This syndrome occurs when small blood vessels that usually respond to cold or stress become too constricted. It causes pallor and often tingling or numbness in the affected areas (often the fingers). However, in a classic Raynaud's syndrome, the fingers would also be expected to turn blue and then red, which does not seem to be the case here.*

*Carpal tunnel syndrome: tingling and numbness in the hand, especially in the ring and little fingers, could be indicative of this condition. However, the thumb and first three fingers are typically more affected, while the little finger is usually left out.*

*Arthritis: joint pain in more than one joint could indicate arthritis. This could also explain some of the other symptoms, but not all.*

*Peripheral neuropathy: This is a disorder of the nerves that can cause pain, tingling, and numbness, but it usually affects the feet more.*

*Polyneuropathy: A disorder that affects multiple peripheral nerves at once. It often causes symptoms of pain, tingling, or numbness in different parts of the body.*

*Please seek medical advice from a doctor or medical professional to determine the exact cause of these symptoms.*

*User*

*Which clinical diagnosis matches the following: Please provide the top 5 diagnoses*

*this is a female patient born in 1967*

*Present symptoms*

*Morning stiffness*

*persists since: more than one year*

*Knee pain*

*Movement: alleviating*

*Laterality: bilateral*

*Intensity: moderate*

*Symptoms not present:*

*Limited mobility of the*

*knee*

*Joint pain in the fingers*

*Pain in more than one*

*Joint*

*Reduced mobility of the*

*Shoulder*

*Shoulder pain*

*Pressure pain in the knee*

*Hip pain*

*Limp*

*Fatigue*

*Reduced mobility of fingers*

*Pressure pain hip*

*Reduced mobility of the spine*

*General muscle pain*

*Pressure painful finger joints*

*Pain in the arm*

*Knee pain, inner side*

*Knee pain, outside*

*Pain in the neck and*

*Neck area*

*Swollen knee*

*Smoker*

*Pregnant*

*Diabetes*

*High blood pressure*

*ChatGPT*

*Considering the above symptoms and excluding some conditions, one could consider the following conditions. However, it is important to emphasize that this evaluation is based only on the information provided and should not be considered a definitive diagnosis.*

*Osteoarthritis: this is a type of arthritis caused by wear and tear of the joints, often in the knees. It typically causes pain and stiffness especially after rest or in the morning. However, since no limited mobility or swelling of the knee is reported, it could be an early or mild form of osteoarthritis.*

*Rheumatoid arthritis: this is an autoimmune disease that causes chronic inflammation and pain in the joints. However, morning stiffness is typically seen in multiple joints and it usually indicates inflammatory arthritis.*

*Patellofemoral pain syndrome: pain is concentrated in the front of the knee and is often aggravated by activities such as climbing stairs, kneeling or sitting with the knees bent.*

*Bursitis: inflammation of the bursa, a small fluid-filled blister that acts as a cushion between bone and soft tissue, could also cause knee pain.*

*Tendinitis: This is inflammation or irritation of a tendon that can cause pain and tenderness near a joint. In the knee, it could affect the patellar tendon, which is known as "jumper's knee."*

*In any case, the patient should see a medical professional for an accurate diagnosis and appropriate treatment.*

*User*

*Which clinical diagnosis matches the following: Please provide the top 5 diagnoses*

*this is a female patient born in 1945*

*Generalized muscle pain*

*has been present for: one month to one year*

*Morning stiffness*

*Tense shoulder muscles*

*Decreased mobility of the shoulder*

*Pressure painful shoulder*

*Laterality: bilateral*

*Symptoms not present:*

*Fatigue*

*Shoulder pain*

*Pressure painful hip*

*Hip pain*

*Upper arm pain*

*Pain in the neck and*

*Neck area*

*Joint pain in the fingers*

*Groin pain*

*Smoker*

*Diabetes*

*High blood pressure*

*Unclear symptoms:*

*Pain in more than one joint*

*ChatGPT*

*The symptoms the patient is experiencing could indicate several conditions. Here are five possible diagnoses that could be considered, although a definitive diagnosis can only be made by a physician.*

*Adhesive Capsulitis (Frozen Shoulder): This syndrome is characterized by gradual, painful stiffness in the shoulder. Symptoms typically occur gradually, worsen over time, and eventually resolve. This can span a period of one to three years.*

*Rotator Cuff Tear: A tear in one of the shoulder tendons can cause muscle pain and limited range of motion, especially in older patients. Symptoms may resemble those of frozen shoulder.*

*Polymyalgia Rheumatica: This inflammatory condition causes muscle pain and stiffness in various parts of the body, usually in the neck, shoulders or hips. Symptoms are worse, especially in the morning. However, it would be unusual if only the shoulders are affected and other areas are not.*

*Arthritis: Arthritis can cause pain, stiffness and Lead to restricted movement, especially in the joints. It could be a form of arthritis that specifically affects the shoulders, but the lack of joint pain in other areas makes this diagnosis less likely.*

*Fibromyalgia: This is a chronic condition that causes widespread muscle pain and tenderness, as well as other symptoms such as fatigue, sleep disturbances and sometimes morning stiffness. However, it is unlikely if the pain is limited to the shoulders and the patient does not report fatigue.*

*A more detailed examination and diagnosis by a physician is necessary to determine the exact cause of the symptoms.*

*User*

*Which clinical diagnosis matches the following: Please provide the top 5 diagnoses*

*this is a female patient born in 1945*

*Present symptoms*

*Pain in more than one*

*joint*

*has been present for: one month to one year*

*Pain on exertion: worsening*

*Abdominal pain*

*Intensity: severe*

*persists since: one month to one year*

*General muscle pain*

*Fatigue*

*Difficulty concentrating*

*Tingling sensation in the leg*

*Location: bilateral, symmetrical*

*Pressure pain of the*

*back muscles*

*Tingling or numbness in the*

*lower leg*

*Smoker*

*Symptoms not present:*

*Morning stiffness*

*Prolonged night sleep*

*Pressure pain of the*

*Leg muscles*

*Diabetes*

*High blood pressure*

*Uncertain symptoms:*

*Pressure painfulness of lower back muscles*

*ChatGPT*

*Based on the symptoms you describe, here are some possible diagnoses. It is important to stress that an accurate diagnosis can only be made by a doctor or medical professional and this is only an orienting list based on the symptoms provided.*

*Fibromyalgia: This is a chronic condition that causes muscle pain and tenderness. In addition to pain and fatigue, symptoms may include problems concentrating, often referred to as "fibro-fog."*

*Polymyalgia Rheumatica: This is an inflammatory condition that causes muscle pain and stiffness in various parts of the body, often in the neck, shoulders and hips. Symptoms are worse, especially in the morning, but fatigue and a general malaise may also occur.*

*Peripheral neuropathy: This could be the cause of the tingling or numbness in the legs. There can be many causes of peripheral neuropathy, including vitamin B12 deficiency, alcohol abuse, and smoking.*

*Irritable Bowel Syndrome (IBS): this could explain the abdominal pain you describe, especially if it is accompanied by other digestive problems such as diarrhea or constipation.*

*Osteoarthritis: this is a common form of arthritis caused by the gradual wearing away of the cartilage in joints. It can cause pain in more than one joint.*

*.In any case, it is important to see a doctor to further investigate these symptoms and get an accurate diagnosis.*

*User*

*Which clinical diagnosis matches the following: Please provide the top 5 diagnoses*

*This is a female patient born in 1959.*

*Presenting symptoms:*

*Pain in more than one*

*joint*

*persists since: more than one year*

*Pain on exertion: mitigating*

*General muscle pain*

*has been present for: more than one year*

*Morning stiffness*

*Tiredness*

*Pressure painfulness of the*

*Leg muscles*

*Tense leg muscles*

*Symptoms not present:*

*Prolonged night sleep*

*Difficulty concentrating*

*Pressure pain in the*

*Back muscles*

*Joint pain in the fingers*

*Abdominal pain*

*tingling sensation in the leg*

*Knots under the skin at one*

*joint*

*Swollen finger joint*

*Reddened facial skin*

*Reduced mobility of the*

*Shoulder*

*Decreased mobility of fingers*

*Hand pain*

*Dry eyes*

*Decreased sex drive*

*Pain behind the eye*

*Pressure painful abdomen*

*Smoker*

*Diabetes*

*High blood pressure*

*ChatGPT*

*Based on the information provided, the following possible diagnoses could be considered:*

*Rheumatoid arthritis: this condition is a chronic inflammatory disorder that affects multiple joints in the hands and feet. Symptoms include painful joints, generalized muscle pain, morning stiffness and fatigue.*

*Fibromyalgia: Fibromyalgia is a disorder characterized by widespread muscle pain and stiffness, often accompanied by fatigue and sleep disturbances. Muscle tenderness is also a typical feature.*

*Polymyalgia Rheumatica: Polymyalgia Rheumatica is an inflammatory disorder that causes muscle pain and stiffness in various parts of the body, most commonly in the neck, shoulders and hips. The stiffness is usually worst in the morning.*

*Chronic Fatigue Syndrome (CFS): CFS is a complex condition characterized by persistent fatigue that is not improved by rest and worsens with physical or mental activity.*

*Osteoarthritis: Osteoarthritis is a degenerative joint disease that damages the joints and can cause pain and stiffness.*

*It is important for this patient to see a medical professional to get an accurate diagnosis, as many of these conditions can have similar symptoms.*

*Further testing and investigation is needed to determine the exact cause of her symptoms.*

*User*

*Which clinical diagnosis matches the following: Please provide the top 5 diagnoses*

*the patient is a female born in 1973*

*Present symptoms*

*pain in more than one joint*

*persists since: more than one year*

*General muscle pain*

*persists since: more than one year*

*Morning stiffness*

*Joint pain in fingers*

*Laterality: bilateral*

*Intensity: moderate*

*Back pain*

*Movement: ineffective*

*Intensity: moderate*

*Regular physical activity*

*Upper back pain*

*Pain in the lower back*

*radiating into the leg: not present*

*Symptoms not present:*

*Prolonged night sleep*

*Fatigue*

*Decreased mobility in*

*several joints*

*Pressure pain in the*

*Back muscles*

*Knots under the skin at one*

*joint*

*Extreme daytime sleepiness*

*Swollen finger joint*

*Abdominal pain*

*Foot pain*

*BMI over 25*

*Difficulty concentrating*

*Slouched posture*

*Pain in the neck and*

*Neck area*

*Pressure pain*

*Toe base joints*

*Reduced mobility of the toes*

*Joint swelling in more than one*

*location*

*Smoker*

*Pregnant*

*Diabetes*

*High blood pressure*

*ChatGPT*

*Based on the information given, the patient could have the following possible diagnoses:*

*Rheumatoid arthritis: This inflammatory autoimmune disease causes pain and swelling in several joints, including the fingers, and can also lead to generalized muscle pain.*

*Fibromyalgia: This is a neurological disorder characterized by widespread muscle pain and stiffness, often accompanied by fatigue and sleep disturbances. Back pain and morning stiffness are also common.*

*Polymyalgia rheumatica: This inflammatory disease causes muscle pain and stiffness in various parts of the body. However, polymyalgia rheumatica usually occurs in people over the age of 50.*

*Ankylosing spondylitis: This is a form of inflammatory arthritis that primarily affects the spine, causing back pain and stiffness. In some cases, other joints, such as the fingers, may also be affected.*

*Systemic lupus erythematosus (SLE): SLE is a chronic autoimmune disease that can affect different parts of the body, including joints and muscles.*

*Please note that this is only a list of possible diagnoses and an accurate diagnosis should be made by a doctor or healthcare professional. Further examination and testing will be necessary to determine the exact cause of symptoms.*

*User*

*Which clinical diagnosis matches the following: Please provide the top 5 diagnoses*

*it is a female patient born in 1950*

*Presenting symptoms:*

*Pain in more than one joint*

*persists since: one month to one year*

*Pain on exertion: relieving*

*Joint swelling in more than one*

*site*

*has existed for: more than one year*

*Joint pain in the fingers*

*Movement: mitigating*

*Laterality: bilateral*

*Intensity: moderate*

*Morning stiffness*

*Swollen finger joint*

*Pressure painful finger joints*

*Laterality: bilateral*

*Lump under the skin on one*

*joint*

*Pressure painfulness:*

*Pressure painful*

*erythematous: not present*

*size change: not present*

*Fatigue*

*Recurrent respiratory infections*

*Recurrent skin infections*

*Foot pain*

*During movement: ineffective*

*Laterality: bilateral*

*Intensity: moderate*

*Knots under the skin on the*

*finger joints*

*High blood pressure*

*Nonexistent symptoms:*

*Pain in the neck and throat*

*Bunion*

*Pale face*

*Lumps under the skin on the*

*Wrist*

*Smoker*

*Diabetes*

*ChatGPT*

*Based on the symptoms present, the patient could have the following possible diagnoses:*

*Rheumatoid arthritis: this condition is an autoimmune disease that causes pain, swelling and stiffness in the joints. Morning stiffness, fatigue, and recurrent infections may also be related to this condition because the immune system is involved.*

*Psoriatic arthritis: This is a type of arthritis that occurs in some people with the skin condition psoriasis. It can cause pain, swelling, and stiffness in the joints. Skin and respiratory infections may indicate psoriasis.*

*Lupus erythematosus (SLE): Lupus is a systemic autoimmune disease that can cause joint pain, swelling, fatigue, and increased susceptibility to infection. It can also cause lumps under the skin.*

*Gout: Gout is a form of arthritis caused by the buildup of uric acid crystals in the joints. It causes severe pain and swelling in the joints, often starting with the big toe.*

*Osteoarthritis: although the symptoms are more consistent with inflammatory arthritis, osteoarthritis should be considered, especially in older patients with joint pain.*

*Please note that this is only a list of possible diagnoses and an accurate diagnosis should be made by a physician or healthcare professional. Further examination and testing will be necessary to determine the exact cause of symptoms.*

*User*

*Which clinical diagnosis matches the following: Please provide the top 5 diagnoses*

*this is a female patient born in 1948*

*Presenting symptoms:*

*Cold finger*

*persists since: more than one year*

*Tingling or numbness in the hand*

*hand*

*Location: unilateral*

*persists since: more than one year*

*Finger pain*

*persists since: more than one year*

*during movement: alleviating*

*Laterality: bilateral*

*Intensity: moderate*

*Pain in the wrist*

*Movement: aggravating*

*Laterality: unilateral*

*Intensity: moderate*

*Tingling or numbness in*

*first three fingers*

*Decreased mobility of the*

*wrist*

*Weakness in the hand*

*Laterality: bilateral*

*Severity: paresis*

*Difficulty with small, fine*

*muscle movements*

*Forearm pain*

*during movement: without effect*

*Intensity: moderate*

*Painful thumb*

*Diabetes*

*High blood pressure*

*Symptoms not present:*

*Smoking*

*ChatGPT*

*Presenting symptoms suggest a number of possible diagnoses:*

*Carpal tunnel syndrome: The patient has several symptoms that could be indicative of carpal tunnel syndrome, including numbness and tingling in the first three fingers, difficulty with fine movements, and weakness in the hand. These symptoms result from compression of the median nerve in the wrist.*

*Raynaud's phenomenon: Cold fingers and pain in the fingers may indicate Raynaud's phenomenon, a condition characterized by narrowing of the small blood vessels in the hands and feet, often triggered by cold or stress.*

*Diabetic neuropathy: In people with diabetes, high blood sugar levels can damage the nerves in the body, leading to a variety of symptoms, including numbness and tingling in the hands and fingers.*

*Arthritis: Pain and decreased mobility in the wrist, as well as pain in the fingers, can be signs of arthritis, an inflammatory condition that affects the joints.*

*Tendinitis or tendonitis: This could also be a possible diagnosis, as pain in the wrist, forearm and thumb can be signs that a tendon is inflamed.*

*These are only possible diagnoses. The exact diagnosis should be made by a doctor or health care professional. Further tests and examinations are needed to determine the exact cause of the symptoms.*

*User*

*Which clinical diagnosis matches the following: Please provide the top 5 diagnoses*

*this is a female patient born in 1953*

*Presenting symptoms:*

*Finger pain*

*persists since: more than one year*

*on movement: without effect*

*Laterality: bilateral*

*Intensity: moderate*

*Decreased mobility of fingers*

*persists since: more than one year*

*Pale fingers*

*persists since: more than one year*

*Joint pain in fingers*

*Movement: increasing*

*Laterality: bilateral*

*Intensity: severe*

*Morning stiffness*

*Knots under the skin on the*

*finger joints*

*pressure painfulness:*

*Pressure painful*

*erythematous: present*

*Size change: present*

*Pressure painful finger joints*

*Laterality: bilateral*

*Pain in more than one*

*joint*

*Pain on exertion: worsening*

*Swollen finger joint*

*Smoker*

*High blood pressure*

*Symptoms not present:*

*Overheated skin over one*

*Finger joint*

*Back pain*

*Fatigue*

*Pain in the neck and*

*neck area*

*Pressure painful knee*

*Bent posture*

*Foot pain*

*Pressure painful hip*

*Hip pain*

*Knee pain*

*Pressure painful*

*Basal joints of toes*

*Joint swelling in more than one*

*Location*

*Swollen hand*

*Pressure painfulness of the*

*Back muscles*

*Diabetes*

*ChatGPT*

*The given symptoms and course suggest several possible diagnoses:*

*Rheumatoid Arthritis: The patient presents with several symptoms that could be consistent with rheumatoid arthritis, including morning stiffness, pain and swelling in multiple joints, and nodules under the skin on the finger joints. Rheumatoid arthritis is a chronic autoimmune disease in which the immune system mistakenly attacks healthy tissue, causing inflammation in the joints.*

*Osteoarthritis (Osteoarthritis): This is a degenerative joint disease that mainly affects older people. It causes pain and stiffness in the joints. In this patient, osteoarthritis could be an explanation for the finger and joint pain and decreased finger mobility.*

*Raynaud's phenomenon: Pale fingers may be a sign of Raynaud's phenomenon, a disorder that causes narrowing of the small blood vessels in the fingers and toes, often triggered by cold or stress.*

*Psoriatic arthritis: this form of arthritis occurs in some people with psoriasis and causes joint pain, swelling and stiffness. The fact that the skin over the finger joints is erythematous (red) could indicate psoriatic arthritis.*

*Gout: This is a form of arthritis caused by a buildup of uric acid crystals in the joints. Symptoms may include pain, swelling and stiffness in the joints, including the fingers.*

*Please note that this list is only possible diagnoses based on the information provided. An accurate diagnosis requires a comprehensive clinical examination and further testing if necessary, and should be made by a physician or healthcare professional.*

*User*

*Which clinical diagnosis matches the following: Please provide the top 5 diagnoses*

*The patient is a male born in 1960*

*Presenting symptoms:*

*Decreased mobility in several jointshas been present for: more than one year*

*Pain in more than one joint*

*has been present for: more than one year*

*Pain on exertion: alleviating*

*Morning stiffness*

*Joint pain in the fingers*

*Movement: mitigating*

*Laterality: bilateral*

*Intensity: mild*

*General muscular pain*

*Pain in the neck and*

*neck area*

*Bunion*

*Weight loss*

*more than 10% of body weight in 6*

*months: not present*

*High blood pressure*

*Symptoms not present:*

*Pressure painful finger joints*

*Lump under the skin on one*

*Joint*

*Swollen finger joint*

*Fatigue*

*Back pain*

*Prolonged night sleep*

*Shoulder pain*

*Extreme daytime sleepiness*

*Foot pain*

*BMI over 25*

*Pressure painful hip*

*Pressure pain in the*

*Back muscles*

*Neck pain*

*Altered nail growth*

*Tense muscles all over the*

*Body*

*Smokers*

*Diabetes*

*ChatGPT*

*Based on the information given, this 1960 male patient could have the following possible diagnoses:*

*Rheumatoid Arthritis: The presence of morning stiffness, pain in multiple joints, and joint pain in the fingers could indicate rheumatoid arthritis. Although this is usually a chronic inflammatory disease that affects the immune system and causes swelling and pain in the joints, pain relief with movement and the absence of swelling or lumps under the skin may also be present.*

*Osteoarthritis (Osteoarthritis): Symptoms could also be indicative of a degenerative joint disease such as osteoarthritis, especially because of decreased range of motion in multiple joints and pain in multiple joints. Fibromyalgia: Although the absence of fatigue and the mitigating effect of exercise on pain are not typical of this condition, the generalized muscle pain could be suggestive of it. Hypothyroidism: although less likely, hypothyroidism can cause muscle pain, morning stiffness, and joint pain. Spondyloarthropathies: these include conditions such as psoriatic arthritis and ankylosing spondylitis, which can cause chronic joint pain and stiffness.*

*Fibromyalgia: Although the absence of fatigue and the mitigating effect of exercise on pain are not typical of this condition, the generalized muscle pain could be suggestive.*

*Hypothyroidism: although less likely, hypothyroidism can cause muscle pain, morning stiffness, and joint pain.*

*Spondyloarthropathies: these include conditions such as psoriatic arthritis and ankylosing spondylitis, which can cause chronic joint pain and stiffness.*

*Please note that this list is only possible diagnoses based on the information provided. An accurate diagnosis requires a comprehensive clinical examination and further testing, if necessary, and should be made by a physician or health care professional.*

*User*

*Which clinical diagnosis matches the following: Please provide the top 5 diagnoses*

*the patient is a female born in 1988*

*Present symptoms:*

*Scaling skin on the*

*outer sides of the arms and the*

*front of the legs*

*Finger pain*

*persists since: one month to one year*

*with movement: alleviating*

*Laterality: bilateral*

*Intensity: moderate*

*Itching on the back of the arms*

*and the front of the legs*

*Morning stiffness*

*Knee pain*

*Movement: mitigating*

*Laterality: bilateral*

*Intensity: moderate*

*Joint pain in fingers*

*Movement: mitigating*

*Laterality: bilateral*

*Intensity: moderate*

*Non-existent symptoms:*

*Hardened and thickened skin on*

*the outside of the arms and the*

*front of the legs*

*Decreased mobility of the fingers*

*Swollen toe joint*

*Swollen finger joint*

*Spots on the arm*

*Smoker*

*Pregnant*

*Diabetes*

*High blood pressure*

*Uncertain symptoms*

*Pressure painful finger joints*

*ChatGPT*

*Based on the symptoms provided, the following diagnoses might be considered:*

*Psoriatic arthritis: The combination of skin changes (such as scaly skin) and joint pain (such as finger pain and knee pain) could indicate psoriatic arthritis. Although the patient does not have hardened or thickened skin, psoriasis may also be characterized by dry, scaly skin.*

*Keratosis Pilaris: A skin condition characterized by rough, scaly patches and itching on the outside of the arms and the front of the legs. This condition is not associated with joint pain, but it could be a coincidence between two different conditions.*

*Rheumatoid arthritis: the symptoms of morning stiffness and joint pain in the fingers could indicate early rheumatoid arthritis, although the absence of joint swelling is not typical of this disease. Joint pain could be relieved with exercise.*

*Osteoarthritis: while less likely in a younger patient, the joint pain and morning stiffness could indicate osteoarthritis, especially if relieved by exercise.*

*Fibromyalgia: A chronic pain disorder that can cause generalized and long-lasting pain in various parts of the body, such as the fingers and knees. Morning stiffness is also a common symptom.*

*Please note that these possible diagnoses are made based on the information provided.*

*An accurate diagnosis requires a complete clinical examination and should be made by a physician or healthcare professional. It is especially important to review "uncertain symptoms" and possibly perform further testing.*

*User*

*Which clinical diagnosis matches the following: Please provide the top 5 diagnoses*

*this is a male patient born in 1994*

*Present symptoms*

*Pain in the lower back*

*Movement: mitigating*

*persists since: more than one year*

*intensity: mild*

*radiating into the leg: not present*

*Ankle pain*

*has been present for: more than one year*

*Movement: alleviating*

*Laterality: bilateral*

*Intensity: mild*

*Swollen fingers*

*persists since: one week to one month*

*Laterality: unilateral*

*Morning stiffness*

*Pain in more than one*

*joint*

*Pain on exertion: mitigating*

*Tense back muscles*

*Muscle tension in the lower*

*back*

*Joint pain in the fingers*

*Movement: mitigating*

*Laterality: bilateral*

*Intensity: mild*

*Pressure painful hip*

*Laterality: bilateral*

*Symptoms not present*

*Altered nail growth*

*Decreased mobility in*

*multiple joints*

*Decreased mobility of the spine*

*Decreased mobility of the fingers*

*Spots on the arm*

*Pressure painful finger joints Swollen toe joint*

*Pain in the arm*

*Difficulty breathing*

*Spots on leg*

*Knee pain*

*Lumps under the skin on a jointmuscle cramps in the back*

*Fatigue*

*Smoker*

*Diabetes*

*High blood pressure*

*ChatGPT: The symptoms provided could indicate a variety of conditions. Here are the top 5 possible diagnoses:*

*Ankylosing Spondylitis: This is a type of inflammatory arthritis that affects the spine and can lead to chronic lower back pain. It is often accompanied by morning stiffness. The fact that the pain is relieved by movement is also typical. However, it would be unusual for the pain in this condition to be relieved by exercise. Rheumatoid arthritis: the morning stiffness and joint pain in the fingers could indicate this autoimmune disease. However, the unilateral swelling of the fingers is rather atypical.*

*Spondyloarthritis: this is a group of inflammatory joint diseases that can cause pain and inflammation, especially in the spine. However, it is common in this condition for pain to be relieved by rest and worsened by activity, which is inconsistent with the symptoms provided.*

*Reactive arthritis: this is a rare type of arthritis that occurs in response to an infection in another part of the body. It could explain the pain in several joints.*

*Osteoarthritis: although this is less common in younger people, the symptoms could indicate early osteoarthritis, especially if relieved by exercise. However, bilateral hip pain is less typical of this disease.*

*It is important to note that these diagnoses are presumptive based on the information provided and an accurate diagnosis requires a comprehensive clinical examination by a physician or health care professional. Further examination and testing may be necessary to make an accurate diagnosis.*

*User*

*Which clinical diagnosis matches the following information: Please provide the top 5 diagnoses*

*this is a female patient born in 1979*

*Presenting symptoms:*

*General muscle painpersists since: more than one year*

*Extreme daytime sleepiness*

*has been present for: one month to one yearFrequent need to urinate*

*has been present for: one month to one year*

*Pain in more than one*

*jointPain on exertion: without effectDecreased mobility inseveral jointsFatigueFluctuating muscle weakness*

*Morning stiffness*

*Tingling sensation in the leg*

*Location: bilateral, asymmetrical*

*Nausea*

*Intensity: mild*

*Pressure pain of the*

*back muscles*

*Pressure painfulness of the lower*

*back muscles*

*Pressure painfulness of the*

*Leg muscles*

*Tingling or numbness in the*

*lower leg*

*Tingling or numbness in the*

*Foot*

*Decreased physical*

*performance*

*Tense leg muscles*

*Diabetes*

*High blood pressure*

*Symptoms not present:*

*Prolonged night sleep*

*Loss of appetite*

*Abdominal pain*

*Difficulty concentrating*

*Smoker*

*Pregnant*

*ChatGPT*

*The patient's symptoms could indicate multiple conditions. Here are the top 5 potential diagnoses:*

*Fibromyalgia: Fibromyalgia is a neurological disorder characterized by generalized muscle pain, fatigue, sleep disturbances, and often symptoms such as memory and mood changes. The exact cause is unknown, but a variety of genetic and environmental factors are thought to play a role. Extreme daytime fatigue, morning stiffness, and pressure-sensitive muscles may be indicative of this condition.Chronic fatigue syndrome: This is characterized by prolonged, debilitating fatigue and other symptoms such as muscle pain, difficulty concentrating, and sleep disturbances.Polyneuropathy: Some of the symptoms, especially tingling or numbness in the legs and feet, could indicate polyneuropathy, a disease of the peripheral nerves that often occurs in diabetes patients.Polymyalgia rheumatica: An inflammatory disease that causes muscle pain and stiffness in the neck, shoulders and hips.Multiple sclerosis: A disease of the central nervous system that can cause symptoms such as fatigue, difficulty with coordination and balance, muscle weakness, and numbness or tingling.*

*It should be noted that the above diagnoses are based on the symptoms provided and that an accurate diagnosis requires a complete examination by a physician. Further testing may be necessary to make a definitive diagnosis.*

*User Which clinical diagnosis matches the following: Please provide the top 5 diagnoses*

*This is a male patient born in 1978*

*Present symptomsJoint pain in the fingers*

*persists since: one month to one year*

*Movement: mitigating*

*Laterality: bilateral*

*Intensity: moderate*

*Morning stiffness*

*persists since: one month to one year*

*Pain in more than one*

*joint*

*Pain on exertion: ineffective*

*Pressure painful finger joints*

*Laterality: bilateral*

*Back pain*

*Movement: aggravating*

*Intensity: moderate*

*Foot pain*

*during movement: alleviating*

*Laterality: unilateral*

*Intensity: mild*

*Regular physical activity*

*Upper back pain*

*Bent posture*

*Knee pain*

*Movement: aggravating*

*Laterality: bilateral*

*Intensity: moderate*

*Symptoms not present*

*Swollen finger joint*

*Decreased mobility of the fingers*

*Lump under the skin at one*

*joint*

*Fatigue*

*Altered nail growth*

*BMI over 25*

*Decreased mobility of the spine*

*Lower back pain*

*Pressure painful hip*

*Pressure painfulness of the*

*Back muscles*

*Smokers*

*Diabetes*

*High blood pressure*

*ChatGPT*

*Based on the given symptoms, the following diagnoses could be considered:*

*Rheumatoid arthritis (RA): RA is a chronic inflammatory disease that affects multiple joints. Symptoms may include pain and stiffness in several joints, especially the hands and fingers, and morning stiffness. However, a confirmed diagnosis requires further medical testing and evaluation by a rheumatologist.*

*Osteoarthritis: This degenerative joint disease occurs when the protective cartilage that cushions the ends of your bones wears away. This can lead to symptoms such as joint pain and stiffness.*

*Ankylosing spondylitis: This is a type of inflammatory arthritis that affects some of the vertebrae in your spine. It can cause severe, chronic pain and discomfort and is often associated with morning stiffness.*

*Psoriatic arthritis: this type of arthritis affects some people who have psoriasis - a condition that causes rapid skin cell growth. Joint pain, stiffness and swelling are the main signs and symptoms of psoriatic arthritis.*

*Gout: Gout is a form of arthritis characterized by periodic bouts of severe joint pain. However, it is more likely to affect individual joints and symptoms often occur suddenly.*

*Please note that these potential diagnoses are based on the symptoms given and that an accurate diagnosis requires a complete medical examination and possibly additional testing. The patient should see a physician for an accurate diagnosis and treatment.*

*User*

*Which clinical diagnosis matches the following: Please provide the top 5 diagnoses*

*this is a female patient born in 1992*

*Present symptoms*

*Rash on the face*

*pruritus: present*

*pushable away: present*

*raised: present*

*persists since: one month to one year*

*Headache*

*laterality: unilateral*

*throbbing: present*

*bending forward: aggravating*

*persists since: less than one day*

*intensity: severe*

*Joint pain in fingers*

*persists since: more than one year*

*Movement: ineffective*

*Laterality: unilateral*

*Intensity: mild*

*Diarrhea*

*persists since: less than one day*

*blood: not present*

*watery: not present*

*Anxiety*

*Impairment of daily life:*

*Mild or moderate impairment*

*Light sensitive eyes*

*Altered nail growth*

*Morning stiffness*

*Spots on the arm*

*Color: livid*

*Pain: absent*

*pruritus: present*

*squeezable: present*

*scaly surface: not present*

*with raised rim and central*

*fading: not present*

*Symptoms not present:*

*Itching inside the mouth*

*Nausea*

*Pupils of different sizes*

*Tremor*

*Recent reduction in*

*Alcohol consumption*

*Pain around the eye*

*Pain behind the eye*

*Fever*

*Decreased mobility of fingers*

*Sores in the mouth*

*Ingestion of opium-containing*

*drugs or medications*

*Decreased mobility of the*

*Neck*

*Abdominal pain*

*Vomiting*

*Weight loss*

*Smoker*

*Pregnant*

*Diabetes*

*High blood pressure*

*Uncertain symptoms:*

*Spots in the mouth*

*Knee pain*

*ChatGPT*

*Based on the information provided, the following diagnoses might be considered:*

*Systemic lupus erythematosus (SLE): SLE is a chronic autoimmune disease that affects multiple organs and systems in the body. Symptoms may include skin rash, joint pain, morning stiffness and increased sensitivity to light.*

*Migraine: A migraine is a type of headache that can cause severe, throbbing pain that often occurs on one side of the head. Light sensitivity is also a common symptom.*

*Rheumatoid arthritis (RA): RA is a chronic inflammatory disease that affects the joints. Symptoms can include pain, swelling and morning stiffness in the joints.*

*Psoriasis: Psoriasis is a chronic skin disease that can cause patches on the skin and changes in nail growth. In some cases, psoriasis may also be accompanied by joint pain (psoriatic arthritis).*

*Rosacea: Rosacea is a chronic skin condition that causes redness and visible blood vessels on the face. It can also cause eye problems, such as increased sensitivity to light.*

*These are only hypotheses based on the symptoms given. For an accurate diagnosis, the patient should consult a physician. It may be necessary to perform additional tests to make the correct diagnosis and begin appropriate treatment.*

*User*

*Which clinical diagnosis matches the following: Please provide the top 5 diagnoses*

*This is a male patient born in 1955*

*Presenting symptoms:*

*Dry mouth*

*persists since: one month to one year*

*Swollen toe joint*

*has been present for: one month to one year*

*Swollen metatarsophalangeal joint*

*Dry eyes*

*Something in the eye*

*Foot pain*

*During movement: without effect*

*Laterality: unilateral*

*Intensity: moderate*

*Pressure painful*

*Metatarsophalangeal joints*

*Laterality: unilateral*

*Reddened skin on the toe joint*

*Pain: present*

*Pruritus: not present*

*Swelling of the face*

*Symptoms not present:*

*BunionReduced mobility of the*

*Metatarsophalangeal joint of big toeSwollen*

*Metatarsophalangeal joint of big toe*

*Lump under the skin at a*

*jointCrooked toe*

*Respiratory arrest during sleep*

*Morning stiffness*

*Fatigue*

*Difficulty swallowing*

*Arm or leg injury*

*Joint pain in the fingers*

*Painful mouth*

*Pain in more than one*

*Joint Swelling in front of the ear*

*Smoker*

*Diabetes*

*High blood pressure*

*ChatGPT Based on the information given, the following diagnoses could be considered:*

*Gout: Gout is a form of arthritis caused by excess uric acid in the body. This can cause painful crystals to form in joints, often in the big toes. Symptoms may include swelling, redness, and severe pain. Sjögren's syndrome: This is an autoimmune disease that affects the glands that produce moisture, such as tear glands and salivary glands. This can lead to dry eyes and dry mouth. Swelling of the face could also be a sign of parotid (one of the major salivary glands) involvement.*

*Angioedema: Angioedema is swelling that occurs deeper under the skin, often around the eyes and lips, and sometimes hands, feet, and throat. It may be caused by an allergic reaction or a hereditary disorder.*

*Arthritis: Arthritis is an inflammation of the joints that can cause pain and swelling. In this case, arthritis could be present due to the patient's age and the presence of pain and swelling in the joints. Drug side effects: Dry eyes and mouth, swelling and pain can also be side effects of certain medications. It is important to note that a thorough examination by a physician is always required for an accurate diagnosis and appropriate treatment.*

*User*

*Which clinical diagnosis matches the following: Please provide the top 5 diagnoses*

*the patient is a female born in 1992*

*Present symptoms*

*Joint pain at ạnderenọrts not mentioned*

*has been present for: one month to one year*

*Movement: mitigating*

*Laterality: bilateral*

*Intensity: moderate*

*Pain in more than onejoint*

*Pain on exertion: mitigating*

*Morning stiffness*

*Decreased mobility in several joints*

*Pressure pain of the Back muscles*

*Pain in the lower back*

*Movement: mitigating*

*intensity: mild radiating into the leg: absent*

*Extreme daytime sleepiness*

*Pain in the arm*

*during movement: mitigating*

*Laterality: bilateral*

*Intensity: mild*

*ymptoms not present*

*PregnantGeneral muscle pain*

*FatigueProlonged night sleep*

*Pressure pain in finger jointsJoint pain in the fingers*

*Difficulty concentrating*

*Tingling sensation in the leg*

*Abdominal painShoulder painMuscle cramps in the backGroin painWeight lossWatery eyesSmokersDiabetesHigh blood pressure*

*ChatGPTBased on the above symptoms, the following diagnoses might be considered:*

*Fibromyalgia: A disorder that causes pain and stiffness in the muscles and ligaments of the body. Other symptoms may include extreme fatigue, sleep disturbances, headaches and mood swings. Although muscle pain may not be present in your patient, fibromyalgia is still a possibility because of the wide range of symptoms and their variability.*

*Rheumatoid arthritis: a chronic inflammatory disease that affects multiple joints in the hands and feet, causing pain, swelling and stiffness. Even if there is no joint pain in the fingers, this condition can still be considered*

*Lupus Erythematosus: A systemic autoimmune disease that can cause inflammation and pain in any part of the body. Symptoms can vary and may include pain, fatigue, joint stiffness and more.*

*Ankylosing spondylitis (ankylosing spondylitis): A form of arthritis that mainly affects the spine, but it can also affect other joints. Symptoms can include chronic pain and stiffness in the lower back and hip, especially in the morning and after periods of inactivity.*

*Sleep apnea: A serious sleep disorder in which breathing stops and starts repeatedly. Extreme daytime sleepiness may indicate this condition. However, an accurate diagnosis should always be made by a medical professional who will take into account the patient's entire medical history and all symptoms. A thorough clinical examination and further testing, if necessary, are often required to make an accurate diagnosis.*

*User*

*Which clinical diagnosis matches the following: Please provide the top 5 diagnoses*

*the patient is a female born in 1968*

*Existing symptoms*

*Decreased mobility in*

*several joints*

*Exists for: one month to one year*

*Pain in more than one*

*jointhas been present for: one month to one year*

*Pain on exertion: worseningJoint pain in fingers*

*Movement: aggravating*

*Laterality: unilateralIntensity: moderate*

*Morning stiffness*

*Regular physical activity*

*Tense muscles all over theBodyBunion*

*Symptoms that are not present:PregnantPressure painful finger jointsKnots under the skin on theFinger jointsOverheated skin over oneFinger jointFatigueBack painFoot pain*

*General muscle pain*

*Swollen finger joint*

*BMI over 25*

*Stooped posture*

*Pressure pain in the*

*Back muscles*

*Altered nail growth*

*Weight loss*

*Pressure painful*

*Toe base joints*

*Curved toe*

*Pain in the temporomandibular joint*

*Chest pain*

*Smoker*

*Diabetes*

*High blood pressure*

*ChatGPT*

*Based on the above symptoms, the following diagnoses might be considered:*

*Rheumatoid arthritis: A chronic inflammatory disease that can cause joint pain, morning stiffness, and decreased mobility. Although some typical symptoms such as swollen finger joints and overheating of the skin over the joints are absent, it could still be an early or less typical manifestation of the disease.*

*Osteoarthritis: a degenerative joint disease more common in older people that can cause pain and stiffness in the joints. The pain usually intensifies with exertion and subsides with rest.*

*Fibromyalgia: A disorder associated with widespread muscle pain and stiffness. Pain may increase with exertion and patients often have a "stiff" posture.*

*Psoriatic arthritis: a type of arthritis that occurs in some people with psoriasis. It causes joint pain and swelling, but lacks the typical skin symptoms.*

*Gout: A type of arthritis that causes pain, swelling and stiffness in a joint, usually the big toe. The mention of "bunions" could indicate gout, although other typical symptoms are absent.*

*However, an accurate diagnosis should always be made by a medical professional who takes into account the patient's entire medical history and all symptoms. A thorough clinical examination and additional tests, if necessary, are often required to make an accurate diagnosis.*

*User*

*Which clinical diagnosis matches the following: Please provide the top 5 diagnoses*

*the patient is a female born in 1957*

*Present symptoms*

*Joint pain in the fingerspersists since: more than one year*

*Movement: mitigating*

*Laterality: bilateral*

*Intensity: moderate*

*Knee pain, inner side*

*persists since: more than one year*

*Movement: mitigating*

*Laterality: bilateral*

*Intensity: severe*

*Morning stiffness*

*Pain in more than one*

*joint*

*Pain on exertion: mitigating*

*Decreased mobility in*

*several joints*

*Swollen finger joint*

*Smoker*

*Symptoms not present:*

*Pressure painful finger joints*

*General muscle pain*

*Altered nail growth*

*Fatigue*

*Spots on the arm*

*Prolonged night sleep*

*Swollen toe joint*

*Back pain*

*Lumps under the skin on a*

*JointSpots on the leg*

*Foot pain*

*Scaly skin over a*

*joint*

*Bunion*

*Pressure painful hip*

*Bent posture*

*Crooked toe*

*Diabetes*

*High blood pressure*

*ChatGPT*

*Based on the above symptoms and factors, the following clinical diagnoses could be considered:*

*Rheumatoid arthritis: This chronic inflammatory disease mainly affects the small joints and can lead to pain, swelling and reduced mobility. Morning stiffness is a characteristic symptom of this disease.*

*Osteoarthritis: this is a degenerative joint disease that often affects older people. It can cause pain and stiffness in the joints, especially in the knees and fingers. Smoking may increase the risk for this disease.*

*Lupus (systemic lupus erythematosus): This is an autoimmune disease that can lead to a variety of symptoms, including joint pain and swelling. However, it is important to note that other symptoms of lupus, such as skin rashes and extreme fatigue, are not mentioned here.*

*Fibromyalgia: A chronic pain disorder that can cause pain and stiffness in multiple joints and muscles. A symptom of fibromyalgia may include morning stiffness.*

*Gout: A metabolic disease that can lead to severe joint pain. Smoking is a risk factor for gout, but not all the typical symptoms of gout, such as severe pain in the big toe, are present here.*

*It is important to note that an accurate diagnosis should be made by a medical professional who will take into account the patient's entire medical history and all symptoms. Further examination and testing may be required to make a definitive diagnosis.*

*User*

*Which clinical diagnosis matches the following: Please provide the top 5 diagnoses*

*The patient is a male born in 1955*

*Presenting symptoms:*

*Pain in more than one*

*joint*

*persists since: one month to one year Pain on exertion: worsening*

*Morning stiffness*

*Decreased mobility in*

*several joints*

*General muscle pain*

*Foot pain*

*on movement: aggravating*

*Laterality: unilateral*

*Intensity: moderate*

*Regular physical activity*

*Smoker*

*High blood pressure*

*Symptoms not present:*

*Joint pain in fingers*

*Pressure painful finger joints*

*Lumps under the skin on the*

*finger joints*

*Fatigue*

*Prolonged night sleep*

*Extreme daytime sleepiness*

*Back pain*

*Swollen finger joint*

*Difficulty concentrating*

*BMI over 25 Slouched posture*

*Weight loss*

*Fever*

*Tense muscles all over the*

*Body*

*Altered nail growth*

*Pressure pain of the*

*back muscles*

*Bunion*

*Diabetes*

*ChatGPT*

*Based on the symptoms reported, the following clinical diagnoses might be considered:*

*Rheumatoid arthritis: a chronic inflammatory disorder that can affect multiple joints in the body, resulting in pain, swelling and limited range of motion. Although finger joints are often affected, this is not always the case. Smoking may increase the risk for this disease.*

*Osteoarthritis (arthritis): A degenerative joint disease caused by wear and tear of joints due to age or overuse. This disease can cause pain and limited mobility in several joints.*

*Polymyalgia rheumatica: An inflammatory disease that can cause muscle pain and stiffness in various parts of the body. Although it usually affects older adults, it could be the source of this patient's symptoms.*

*Fibromyalgia: A condition that can cause pain and stiffness in various joints and muscles. It is also associated with symptoms such as general muscle pain and morning stiffness.*

*Gout: A metabolic disease that can lead to severe joint pain. Smoking is a risk factor for gout. However, not all the typical symptoms of gout, such as severe pain in the big toe, are present here.*

*It is important to emphasize that only a physician or health care professional can make an accurate diagnosis based on a comprehensive clinical evaluation, which may include laboratory and imaging tests. Please see a* physician for an accurate diagnosis and treatment.
